# Effect of negative pressure therapy on the treatment response to scar thickness and viscoelasticity

**DOI:** 10.3389/fbioe.2024.1353418

**Published:** 2024-04-22

**Authors:** Wei-Cheng Shen, Hsu-Tang Cheng, Yih-Kuen Jan, Ben-Yi Liau, Chang-Wei Hsieh, Jian-Guo Bau, Chien-Cheng Tai, Chi-Wen Lung

**Affiliations:** ^1^ Department of Creative Product Design, Asia University, Taichung, Taiwan; ^2^ Division of Plastic and Reconstructive Surgery, Department of Surgery, Asia University Hospital, Asia University College of Medical and Health Science, Taichung, Taiwan; ^3^ Department of Food Nutrition and Health Biotechnology, Asia University, Taichung, Taiwan; ^4^ Rehabilitation Engineering Lab, Department of Kinesiology and Community Health, University of Illinois at Urbana-Champaign, Urbana, IL, United States; ^5^ Department of Automatic Control Engineering, Feng Chia University, Taichung, Taiwan; ^6^ Department of Electrical Engineering, National Dong Hwa University, Hualien, Taiwan; ^7^ Department of Agricultural Technology, National Formosa University, Yunlin, Taiwan; ^8^ School of Public Health, Taipei Medical University, Taipei, Taiwan

**Keywords:** scar therapy, ultrasound, indentation system, scar thickness, scar viscoelasticity

## Abstract

Patients with scars face a grave threat to their mental and physical health. Negative pressure has been used for scar therapy in medical care and provides a microenvironment conducive to scar healing while stimulating cell regeneration. Negative pressure may disrupt scar tissue regeneration when the pressure is too high or too low, so finding a suitable negative pressure is important. We hypothesized that different negative pressure magnitudes would affect scar tissue properties differently. This research aimed to provide practical recommendations for scar therapy. This study used three negative pressures (−105 mmHg, −125 mmHg, and −145 mmHg) to compare scar material properties. We measured scar tissue thickness and viscoelasticity with a motor-driven ultrasound indentation system. According to the results of this study, scar thickness is most effectively reduced at a negative pressure of −105 mmHg. In comparison, scar viscoelasticity continuously increases at a negative pressure of −125 mmHg. Negative pressure therapy can be recommended to scar care clinics based on the results of this study.

## Highlights

1. This study investigates the magnitude of effective negative pressure in scar treatment.
2. Scar thickness is most effectively reduced with −105 mmHg, and scar viscoelasticity is most steadily increased with −125 mmHg in clinical negative pressure therapy.
3. The stress‐strain curve of the toe regions showed a significant decrease after negative pressure therapy at −125 mmHg compared to −145 mmHg in viscoelasticity.

## 1 Introduction

**FIGURE 1:**
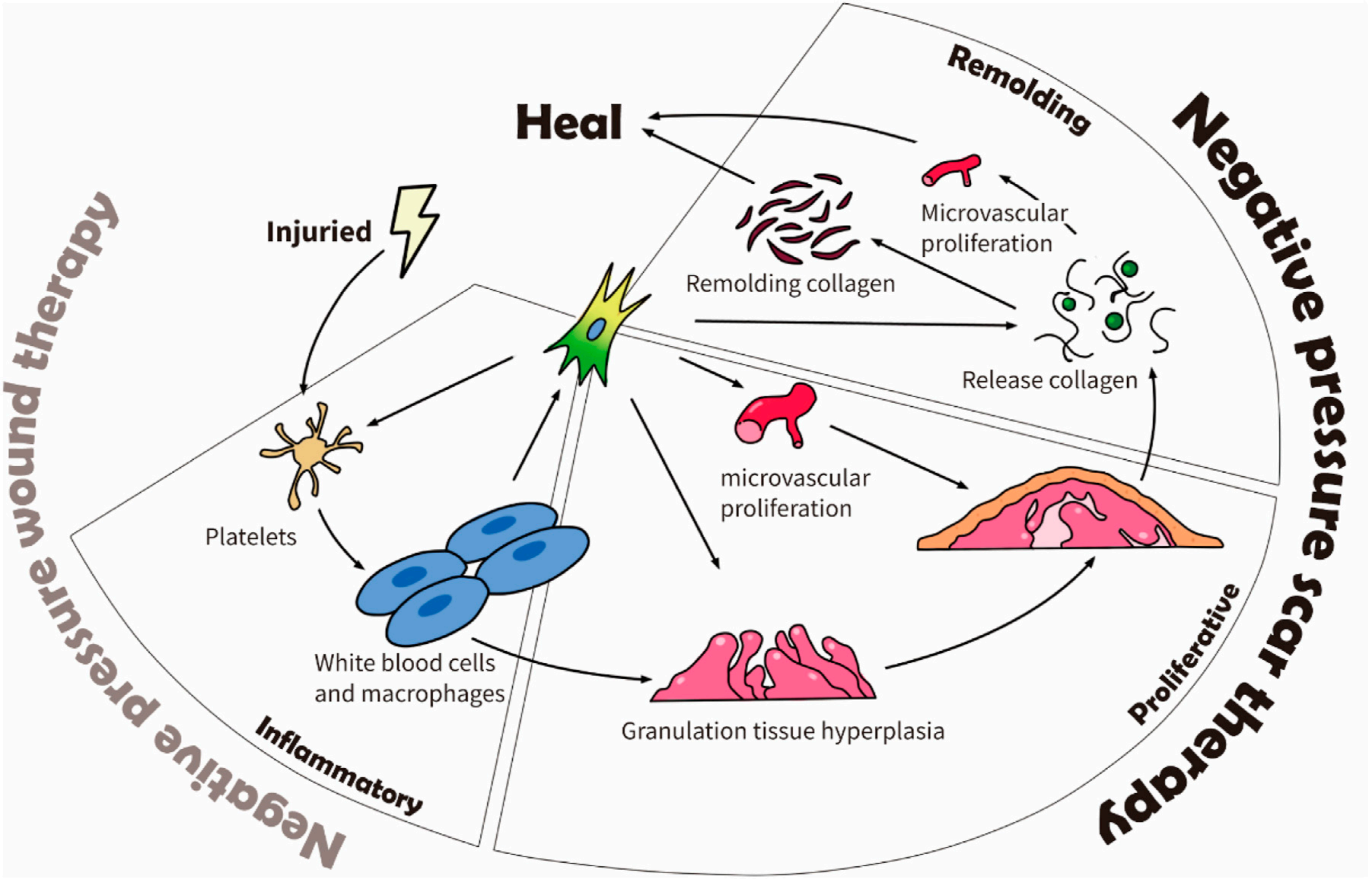
Physiological factors affecting scar healing.

Scars affect people everywhere and often impact patients’ quality of life. Three to four people out of one thousand develop a scar from one or more wounds (Jørgensen et al., 2013). Scars can highly impact the patient psychologically and physically (Brewin and Homer, 2018). For example, Brown et al. demonstrated that scars significantly affect patients’ social functioning (82%), emotional wellbeing (76%), and physical comfort and functioning (59%) (Brown et al., 2008). The physical discomfort effects of a scar include dryness, itching, tenderness, and pain, whereas its functional effects include altered viscoelasticity (Jourdan et al., 2019). The process from the wound to complete scar healing has four consecutive stages: inflammation, proliferation, remodeling, and maturation. It takes 3–5 days after the wound to enter the proliferation stage (Diegelmann et al., 1981), and the scar tissue continues to remodel from 3–6 months after the injury until 12 months (DeJong et al., 2020). Figure 1 shows the scar healing stages and the physiological factors involved.

A hypertrophic scar results from excessive collagen deposition, which results in an exaggerated wound-healing response and a progressive increase in collagen synthesis (Doillon et al., 1985). Such scars are characterized by increased thickness, itchiness, and pain will persist after an injury (Bombaro et al., 2003). Keloids and hypertrophic scars can be reduced by combining injected medicine, postoperative care, and alternative approaches (Son and Harijan, 2014). The goal of scar treatment is to obtain a flat scar with less fibrosis and scar contraction (Son and Harijan, 2014). Symptoms of scars are also affected by how they are treated after recovery. The healing process can take up to 6–12 weeks, and even scars occlusive to the wound surface may require this period before tissue remodeling occurs (Jourdan et al., 2019).

Studies have shown that apoptotic processes can help reduce scar thickness (Darby et al., 2016). Apoptosis is a natural process that eliminates unnecessary cells and tissues without triggering an inflammatory response. This process prevents excessive accumulation of endothelial cells remaining in fibroblasts, which helps flatten the scar tissue (Greenhalgh and biology, 1998). If a scar is damaged again during the healing process, apoptosis will be delayed, and excessive collagen deposits will build up (Torkian et al., 2004; Feng et al., 2020). Currently, a scar is understood to be soft tissue composed primarily of collagen and myofibroblasts that seals a wound (Feng et al., 2020). The collagen and elastin fibers in the scar are thinner, fragmented, and more disorderly than the normal skin (Chen et al., 2015). In addition, the fragmented and disorganized collagen and elastin fibers can cause the gap between fibers to thicken and increase scar thickness (Feng et al., 2022).

Scars are often weaker and have less tensile strength than healthy skin (Shumaker et al., 2012). Scar contracture occurs due to disorderly fibers interfering with fiber sliding, causing decreased extensibility and increased tension around the scar (Corr and Hart, 2013; Feng et al., 2022). Due to a lower tensile strength, the scar tissue is more vulnerable to tensile damage (Tan and Wu, 2017).

Increasing dermal regeneration and modifying fibroblast alignments may improve tensile strength performance (Almine et al., 2012; Hosseini et al., 2022). Atkinson et al. discovered that aligning scar fibers can increase the tensile strength of scars by 20%–80%, and the increased tensile strength helps resist tension damage and accelerates scar healing (Atkinson et al., 2005). The tensile strength is expressed mechanically as a stress–strain curve, while viscoelasticity deformation is considered skin deformation under stress (Hussain et al., 2013). Viscoelasticity refers to the nonlinear relationship between the applied force and the deformation of soft tissues and has been used for research purposes (Hendriks, 1969; Jan et al., 2013). Estimating scar viscoelasticity may provide new insight into changes in scar mechanical properties on the progression of scar healing and treatment outcomes (Feng et al., 2022).

Scar treatments can be categorized into noninvasive and invasive methods. Common noninvasive treatments include silicone gel sheeting, compression therapy, massage, and pressure therapy. The negative pressure therapy (NPT) in this study is one kind of noninvasive treatment. The common invasive treatments involve intralesional corticosteroid injections, surgery, and radiotherapy (Kim, 2021). Interventions that alter the scar thickness and viscoelasticity are common in physical therapy (e.g., massage, silicone gels, and pressure therapy) (Poetschke and Gauglitz, 2016). Although these treatment methods can improve local scarring, certain limitations remain (Heppt et al., 2015). Due to practical medical principles, scar treatment techniques can be combined safely and synergistically with optimal patient results (Zaleski-Larsen et al., 2016). Therefore, developing a simple and feasible treatment method to improve the healing quality of scars is of practical significance.

**FIGURE 2:**
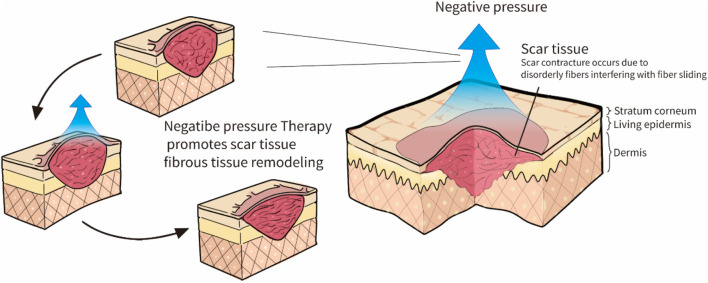
Negative pressure therapy may benefit scars: Conceptual diagram.

Scars may benefit from NPT. Many scholars have used negative pressure to slow the formation of scars, and the technique has been shown to be effective (Zwanenburg et al., 2021). The NPT may increase microcirculation and reduce local skin stiffness (Nicoletti et al., 2017; Wilkes et al., 2012; Al-Bedah et al., 2019). Various mechanisms have been proposed to explain the potential benefits of NPT (Grinnell et al., 1999; Melis et al., 2002; Chen et al., 2015; Mehta and Dhapte, 2015; Moortgat et al., 2016; Al-Bedah et al., 2019). NPT can produce a massage effect when suction forces are applied to tissues (Mehta and Dhapte, 2015), and stretching the local skin prompts a rapid parallel rearrangement of collagen (Chen et al., 2015). The NPT may release the mechanical stress associated with scar retraction by rearrangement of the collagen and elastin, thus inducing apoptosis to decrease scar thickness (Grinnell et al., 1999; Moortgat et al., 2016). Additionally, related research demonstrated that NPT could improve burn scar viscoelasticity, but its quality was poor compared to influencing factors (Moortgat et al., 2016). Both routine and polarized light microscopy can observe negative pressure stretching forces during NPT, resulting in histological and histomorphological changes that rapidly realign fiber tissue (Melis et al., 2002). Figure 2 illustrates how the concept is generally conceptualized. Various magnitudes are currently used in NPT for treating scars (Fraccalvieri et al., 2011), but the lack of standardized application guidelines hampers the use of NPT in scar treatment (Cirocchi et al., 2016). When NPT was increased at intervals of −35 mmHg in animal research of magnitude under −10 to −175 mmHg, blood flow gradually increased until it reached a 90% increase at −80 mmHg (Borgquist et al., 2010).

However, the studies of these treatments often focus on wound healing (inflammation stage) and reduce attention once scarring has occurred (Borgquist et al., 2010). Therefore, the effectiveness of NPT magnitude after scar tissue appears remains unclear. The mechanical dilation of superficial capillaries caused by higher negative pressure is thought to lead to ecchymosis and eventual rupture of these vessels, leaving erythema (Zhao et al., 2009). Negative pressure is one of the intensity factors that change the cupping therapy and may influence the effectiveness of NPT (Lowe, 2017). To improve scar healing while preventing new blood vessels from rupturing, a testing program is needed to clarify the NPT magnitude.

Overall, NPT is considered a possible treatment for scars due to its ability to relieve scar contracture and allow for the rearrangement of scar fibers. However, how NPT’s negative pressure affects scar thickness and viscoelasticity has not been extensively studied. We hypothesized that various NPT magnitudes would cause different treatment responses to the scar. The research aimed to provide a basic skin care plan and practical recommendations for the mechanical properties of scar therapy. The research would check the different treatment magnitudes of NPT in scar tissue and the effect of scar thickness and viscoelasticity on a basic skin care plan and provide practical recommendations for the mechanical properties of scar therapy.

## 2 Materials and methods

### 2.1 Research design

**FIGURE 3:**
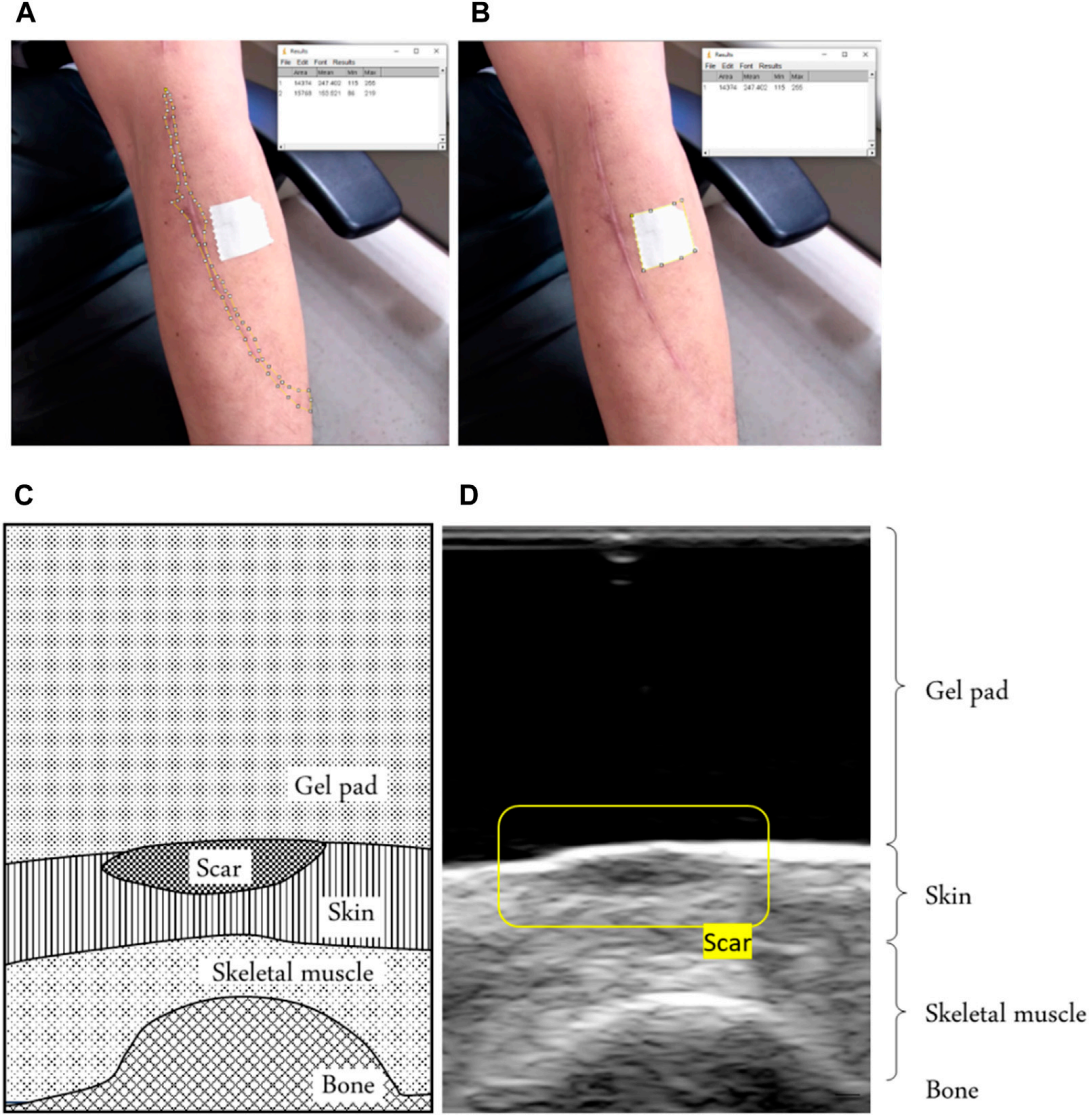
**(A)** Example of defining the scar area by using the reference object area in the image and **(B)** the scar area in the image. Using ImageJ software, we calculated the scar area, using a tape of 20 mm^2^ around it as a reference. **(C)** An example of a scar in a B-mode ultrasound image of a schematic diagram showing the scar. **(D)** B-mode ultrasound signals showing the scar.

Most clinic wound NPTs currently being conducted are medical cases or animal experiments (Zhao et al., 2009; Scalise et al., 2015; Boriani et al., 2018). A limitation of this study was that the subjects were recruited in outpatient clinics, and most of them were injury victims in their early stages of healing. Fewer patients meet the recruiting criteria. Therefore, this study refers to Cupping’s NPT comparative study. According to other research, 15 subjects were invited per group (Wang et al., 2020; Tehseen et al., 2022). Because the NPT was divided into three treatment groups (−105, −125, −145 mmHg), 45 subjects were recruited. The recruitment targets are people with hypertrophic scars assessed by clinic doctors. The subject ethnicity and other body information were obtained by self-description. All participants signed a consent form before participating. The inclusion criteria were adults aged at least 18 years old. The wound must have been present for at least 21 days to ensure it was scar tissue (Sorg et al., 2017). Subjects had not received any other treatment plans in the short term, such as pressure garments, silicone scar gel, intra-scar steroid injections, *etc.* The exclusion criteria included incomplete wound healing, edema, or scars in limited sites such as fingertips. Individuals with diabetes or decubitus were also excluded from this study. Furthermore, as the contraction resulting from the scar can create tension on the surrounding skin, it may influence the viscoelasticity of the scar (Flynn and McCormack, 2008). To avoid the impact of skin tension around scars on viscoelasticity measurements, the scar area (greater than 2.5% of body surface area) was used primarily as a criterion for selecting and recruiting subjects. The skin is the largest organ in the human body. The skin area of men with normal BMI is approximately 1.88 ± 0.14 square meters, and that of women is approximately 1.66 ± 0.20 square meters (Verbraecken et al., 2006). Because the treatment equipment used in this study cannot attach to all scar areas, this article will use the thickness and viscoelasticity of the treatment area, which can be evaluated based on the internal structure of the soft tissue, as a main criterion for scar treatment evaluation. Patients were informed that they could terminate the trial at any time if they felt ill during the treatment. The scar area is calculated using ImageJ analysis software version 1.34e (National Institutes of Health, Bethesda, MD) from the reference object attached to the body surface (Figure 3). Because participants were recruited from clinic visits, scar type and injury location were randomized. The participants were randomly assigned to all three treatment groups using a simple random sampling procedure. A total of 36 subjects were evaluated at the end of the study. The negative pressure of −125 mmHg is the clinically recommended NPT (Gupta et al., 2016). The interval between increments in negative pressure therapy is commonly 20 mmHg (Anesäter et al., 2011; Malmsjö et al., 2011). Therefore, this study’s NPT group was established based on the increased and decreased intervals of negative pressure. Thickness and viscoelastic data from each subject were recorded three times in a relaxed state at every step. Each subject received only one treatment. The Research Ethics Committee of the China Medical University & Hospital, Taichung, Taiwan (CMUH110-REC3-086) approved the study.

### 2.2 Participants

Ultrasonic propagation properties of tissues are widely reported to be sensitive to the alterations of tissue compositions and structure (Huang et al., 2007; Lee et al., 2022). Scars are identified in the site and thickness by B-mode ultrasound image recognition. A linear ultrasound probe with 12 MHz frequencies (5–12 MHz, 128 elements, 39 mm array footprint, Telemed, Vilnius, Lithuania) was attached to a PC-based ultrasound system (ArtUs EXT-1H scanners, Telemed, Vilnius, Lithuania). The research reliably documented scar tissue thickness with 12 MHz ultrasound equipment (Li et al., 2013).

A standoff gel pad was used to cushion and uniform the squeezing effect of the ultrasound probe above the scar during the test. We can identify scars through ultrasound images because a scar has a smaller strain value than normal skin (Aya et al., 2014). Therefore, under the coverage of a standoff gel pad, it shows a higher thickness than the surrounding skin, as shown in Figure 3.

### 2.3 Experimental procedures

**FIGURE 4:**
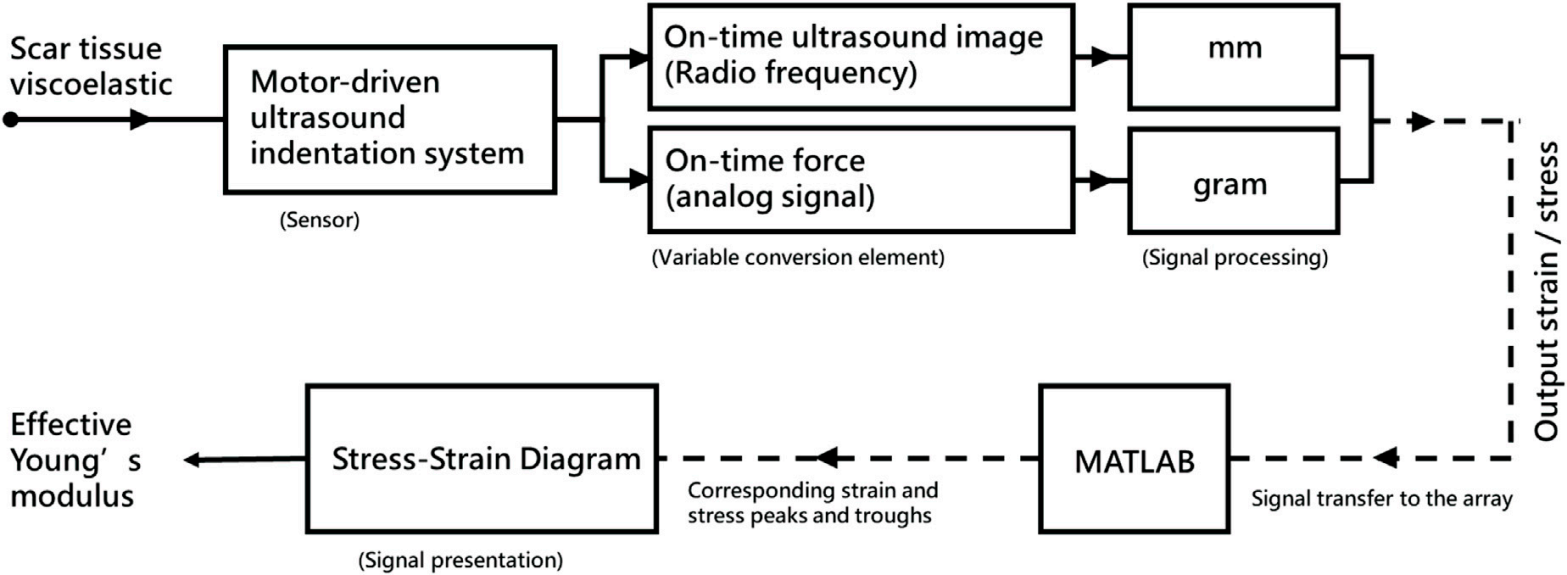
Diagram of the motor-driven ultrasound indentation measurement system.

Scar viscoelasticity was measured by indentometric curves, which responded to pressure loading, while creep and stress relaxation processes were analyzed quantitatively following methods described in previous research (Jan et al., 2013; Pusparani et al., 2022). Each test consisted of five loading cycles. During loading and unloading, the thickness of the scar tissue changes, and the ultrasound echo signal displays and records the thickness and deformation of the real-time soft tissue layer (Jan et al., 2013). A motor-driven ultrasound indentation system was developed to measure the scar tissue’s viscoelasticity. This system includes an ultrasound and load cell that measures the force-deformation responses of the scar tissue. The ultrasound echo signal determines the thickness and on-time deformation of the soft tissue layer. A compressive load cell is connected in series with the ultrasound transducer to record the corresponding force response (Zheng Y. and Mak A. F. J. I. T. O. R. E., 1999). The system uses 12 MHz frequencies (5–12 MHz, 128 elements, 39 mm array footprint, Telemed, Vilnius, Lithuania) attached to a PC-based ultrasound system (ArtUs EXT-1H scanners, Telemed, Vilnius, Lithuania) with a 49-N load cell (Model UKA-E-005, Li-Chen Measure Co., Ltd., Kaohsiung, Taiwan) in series applied to indent the soft tissue. The ultrasonic signal is collected to extract the tissue’s initial thickness and force-deformation responses, as shown in Figure 4.

**FIGURE 5:**
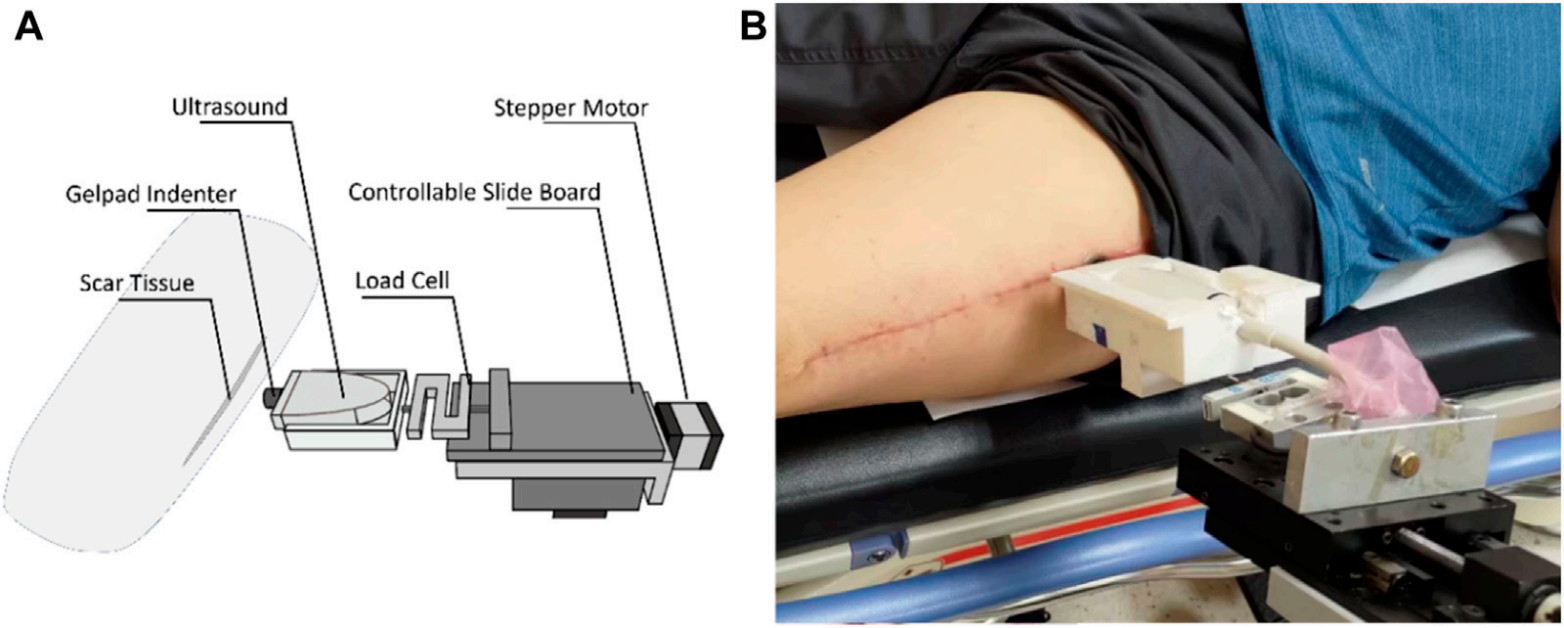
An example of a scar in a B-mode ultrasound image. **(A)** Schematic diagram showing the scar and **(B)** B-mode ultrasound signals showing the scar.

The sampling rate of the image frame and force data were recorded at 22.5 Hz and 100 Hz with a DAQ data acquisition device (USB-6218, National Instrument, Austin, TX, United States of America). In this indentation system, a stepper motor (Model TL-SL1010-X, Tanlian Electro Optics Co., Ltd., Taoyuan, Taiwan) and a stepper motor driven (Model TL-1T, Talian Electro-Optics Co., Ltd., Q17 Taoyuan, Taiwan) with a 1600 micro stepper per revolution, with a step travel of 0.000625 mm and a total travel of 50 mm. The adopted to accomplish an automatic cyclic indentation instead of a manual operation. A standoff gel pad was mounted on the ultrasound transducer probe with a standoff holder (coupling medium, cylinder with 4.5 mm radius and 20 mm thickness, Aquaflex ultrasound gel pad, Parker Laboratory, Orange, NJ) (Jan et al., 2013). The equipment diagram is shown in Figure 5.

**FIGURE 6:**
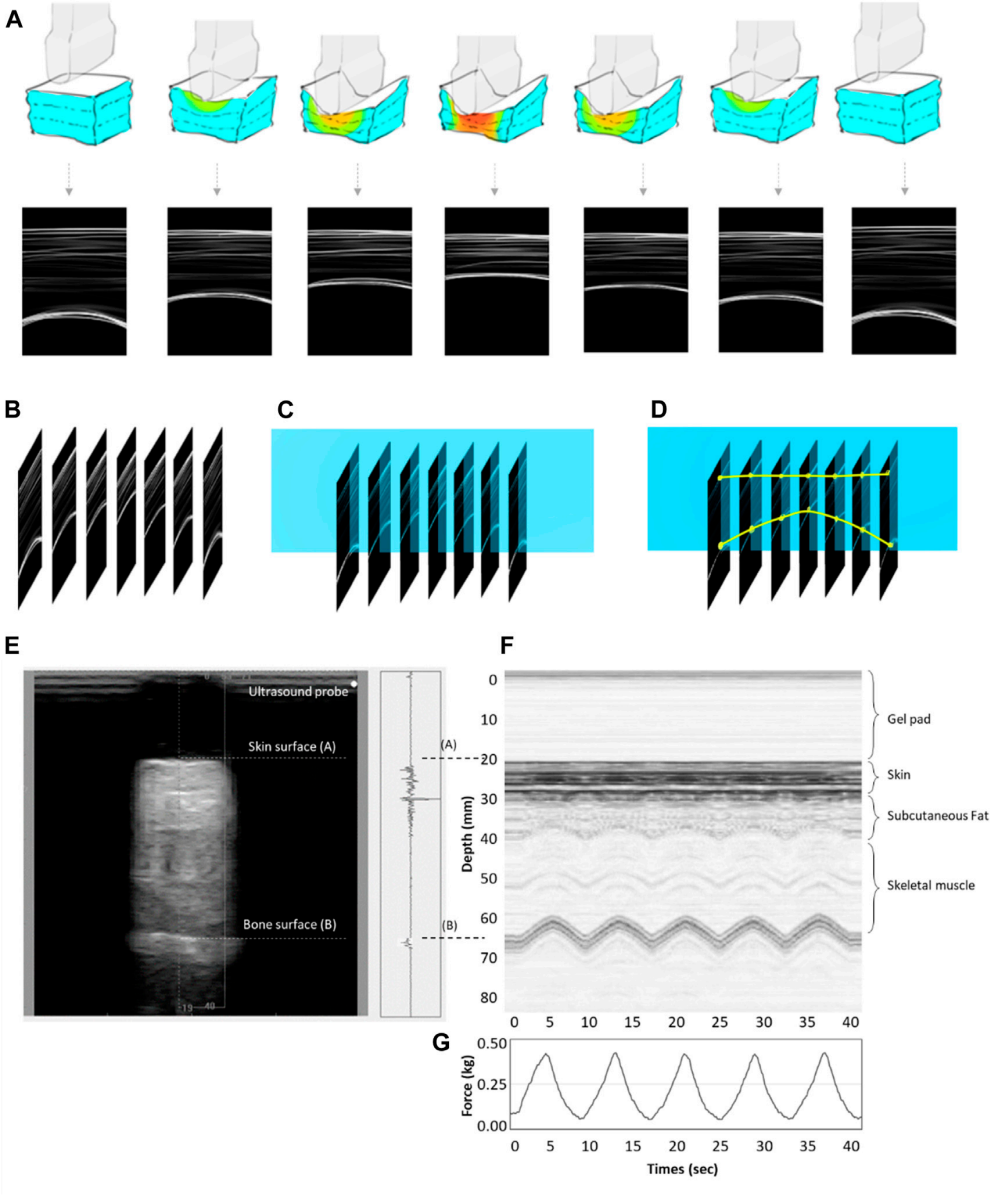
**(A)** The concept map of the motor-driven ultrasound indentation system that compresses the tissue. **(B)** B-mode ultrasound image time axis array. **(C)** Time-lapse image extraction of ultrasound beam data changes. **(D)** A soft tissue anchor point compression transformation of the time axis diagram. **(E)** An example of indentation data obtained from the soft tissue on the scar using the tissue ultrasound palpation system. It shows the M-mode ultrasound signals. The first echo is associated with the ultrasound transducer–skin interface, while the second represents the tissue–bone interface. The thickness of the soft tissue is calculated from the distance between the first and second echoes. **(F)** The real-time ultrasound signals reflected from the tissue–bone interface. **(G)** The time-series information of force under cyclic loadings.

Subjects were asked to lie supine on a hospital bed. Before the measurement, the ultrasound indentation apparatus comprised a 9 mm gel pad diameter and the indenter. After a preload force of less than 0.5 N was applied to the skin perpendicular to the underlying bone, the indent compress was set to 20% of the total subcutaneous soft tissue thickness from real-time ultrasound images (Hayes et al., 1972; Zheng Y. and Mak A. J. J. O. B. E., 1999). Every cyclic load of 40 s was applied with approximately 8 s per loading cycle (Figure 6). Variables related to strain are adjusted based on individual subjects’ characteristics immediately before testing. As the area where the probe contacts the scar is fixed, the stress can be calculated after the load cell has recorded it (Zheng Y. and Mak A. J. J. O. B. E., 1999). The strain in this study was set around 2–4 mm per 4 s, and the response force was 400–600 g.

The effective Young’s modulus (***E***) is a traditional material constant to quantify the elastic properties of soft tissues (Hayes et al., 1972; Zheng Y. and Mak A. J. J. O. B. E., 1999). According to the Egorov et al. research, certain tissue types exhibit similar degrees of nonlinearity regarding effective Young’s modulus, while others exhibit varying degrees (Egorov et al., 2008). Therefore, an effective Young’s modulus can be used to determine whether the structure of soft tissue has changed after scar treatment. We followed the other research with regard to segmentation and performed different compressive strain rates (Ayyildiz et al., 2015). Microstructural composition is complicated (Joodaki and Panzer, 2018), and the different compositions may show the different lead microstructures in the viscoelastic mechanical response of skin tissue. The equation used to extract ***E*** is:

$$E=\frac{\left(1-v^{2}\right)}{2a\cdot \;k\left(v,\frac{a}{h}\right)}\;\cdot \frac{P}{w}$$ (1)

where **v** is the Poisson’s ratio; **a** is the indenter radius; **k** is a scaling factor dependent on the Poisson’s ratio, indenter radius, and tissue thickness; **h** is the soft tissue thickness; ***P*** is the force of pressure loading (indentation); and **w** is the depth of indentation. Generally, 0.45 has been used as the Poisson’s ratio for biological soft tissues, and the radius of the indentor, that is, the ultrasound transducer, was 4.5 mm (Zheng Y. and Mak A. J. J. O. B. E., 1999). The **k** value was obtained from the information extracted from Hayes et al. (1972).

In this study, the data image processing software MATLAB R2020b (MathWorks Inc., MA, US) is used to convert the ultrasonic value and the pressure using the above formula to obtain the elastic coefficient E for analysis. According to Egorov et al., certain tissue types exhibit similar degrees of nonlinearity regarding effective Young’s modulus, while others exhibit varying degrees (Egorov et al., 2008).

**FIGURE 7:**
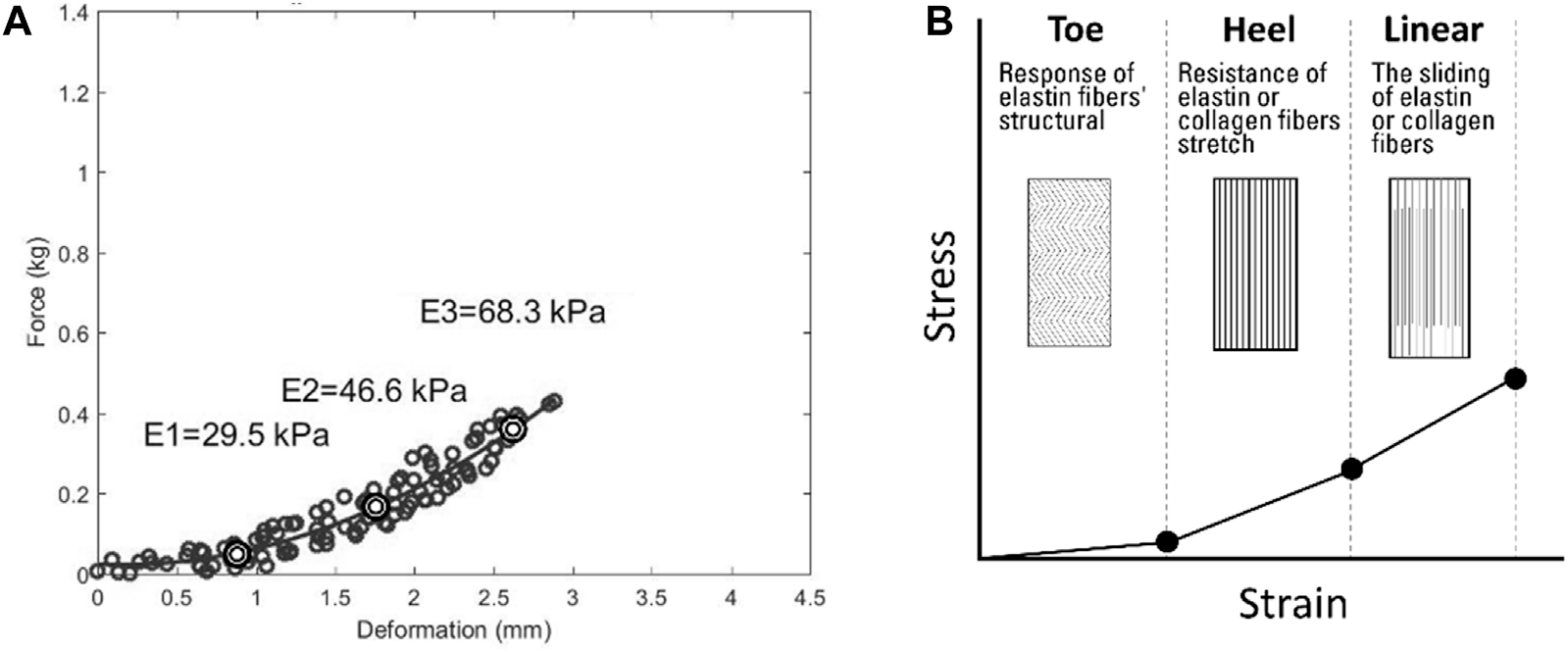
The process of identifying the viscoelasticity value of scar skin. **(A)** Deformation with force showing the viscoelasticity. **(B)** Schematic diagram showing the viscoelasticity. E1, approximately 5% of the initial tissue thickness as the toe region’s viscoelasticity; E2, approximately 10% of the initial tissue thickness as the heel region’s viscoelasticity; E3, approximately 15% of the initial tissue thickness as the linear region’s viscoelasticity.

As shown in Figure 7A, due to its microstructural composition, the mechanical response of skin tissue is highly nonlinear (Joodaki and Panzer, 2018). The microstructural composition of skin tissue is complex, and different compressive strain rates may change its viscoelasticity by altering the leading microstructures in the viscoelastic mechanical response (Joodaki and Panzer, 2018). Based on other research, the segmentation was performed at different compressive strain rates (Ayyildiz et al., 2015). The stress–strain curve of the skin of collagenous tissues is J-shaped and usually divided into the toe region (***E1***), the heel region (***E2***), and the linear region (***E3***) (Fratzl and Weinkamer, 2007; Aziz et al., 2016; Sharabi, 2022), as shown in Figure 7B. In the toe region (***E1***), the skin is relatively soft, and much of the structural response of the skin is carried by elastin components because collagen fibers are slack and non-load-bearing (Joodaki and Panzer, 2018). In the heel region (***E2***), elastic fibers begin to stretch and realign in the direction of the applied force. As the stress–strain curve progresses, collagen in the gap regions begins to resist deformation. In the linear region (***E3***), collagen fibrils have already realigned. The sliding of elastin or collagen causes deformation under stress (Aziz et al., 2016; Sharabi, 2022).

### 2.4 Data analysis

The subjects lay on a ward bed to avoid altering mechanical scar properties through posture. The cup applies negative pressure to the scar, and a sputum extractor (TC-2000V, Taiwan Fukang Assistive Device Leasing Co., Ltd., Taiwan) controls the magnitude of the negative pressure. Before the NPT, each participant’s scar thickness and viscoelasticity were measured. To avoid blisters during cupping therapy, the duration was 10 min, which is considered a short-term effective duration for NPT (Wang et al., 2020). The participant’s scar thickness and viscoelasticity were measured again after NPT. The same expert researcher measured scar thickness and viscoelasticity to avoid inter-observer variability. Although NPT ranges from −75 to −225 mmHg have been reported (Borgquist et al., 2010), the magnitude of −125 mmHg was chosen based on a previous study (Astasio-Picado et al., 2022) to provide the best wound-healing environment for granulation tissue growth (Zhu et al., 2021). Therefore, this study used −125 mmHg as the base magnitude. To prevent new microvessels from being damaged by negative pressure magnitudes greater than −150 mmHg (Zhao et al., 2009), we refer to related studies to determine the 20 mmHg increments and decrements applied (Anesäter et al., 2011).

### 2.5 Statistical analysis

The differences between pre-treatment and post-treatment in the scar tissue thickness and three viscoelasticities (***E1***, ***E2***, and ***E3***) were examined by using the *t*-test. For the *post hoc* comparisons, a one-way repeated measures analysis of variance (ANOVA) was used to compare the ratio of pre-treatment and post-treatment between different NPT magnitudes and determine whether the main effect exists. In addition, Pearson’s correlation was used to examine the main effects of the NPT magnitude. The significance level was set at *p* < 0.05. SPSS version 22 (Version 22, IBM, Armonk, NY, United States) was used to implement all statistical tests.

## 3 Results

### 3.1 Effect of air insole on PPG

Following the withdrawal of some invited participants, 36 subjects were enrolled in this study. This study ultimately recruited 17 women and 19 men. There were 11 subjects in the −105 mmHg magnitude group, 13 subjects in the −125 mmHg magnitude group, and 12 subjects in the −145 mmHg magnitude group included in this study. The demographic data of the three groups are shown in Table 1, which lists the amount of scar type and percentages of cause of injury: accident, 44.4%; abrasions, 41.6%; and burns, 14.0%. Total scar locations were torso = 13.9%, upper limbs = 52.7%, lower limbs = 33.4% (−105 mmHg: torso = 18.2%, upper limbs = 36.4%, lower limbs = 45.5%; −125 mmHg: torso = 15.4%, upper limbs = 46.1%, lower limbs = 38.5%; −145 mmHg: torso = 8.3%, upper limbs = 75.0%, and lower limbs = 16.7%). The study included several types of scars. The surgeries in this study include open wound suturing or surgeries to treat conditions like tumors. Abrasions in this study referred to injuries caused by wear and tear, usually resulting from accidents like falls or traffic collisions. Burn types in this study exclusively involved hydrothermal burns. Among the insect bite cases in this study, scarring occurred only in the case of an allergic reaction. The statistics are shown in Table 2.

**TABLE 1:** The body information of subjects in three groups based on NPT magnitudes.

	Magnitude									One-way ANOVA *p*-value	Fisher least square difference (LSD) post hoc		
	−105 mmHg (mean ± SE)			−125 mmHg (mean ± SE)			−145 mmHg (mean ± SE)				−105 mmHg vs. −125 mmHg	−105 mmHg vs. −145 mmHg	−125 mmHg vs. −145 mmHg
Age	35.1	±	11.5	39.6	±	22.3	50.8	±	17.4	0.208	0.623	0.089	0.213
Body height (cm)	167.0	±	9.2	164.0	±	7.6	164.3	±	2.8	0.677	0.415	0.486	0.938
Body weight (kg)	64.7	±	16.8	58.0	±	7.8	64.8	±	11.7	0.453	0.289	0.999	0.289
BMI	22.9	±	3.8	21.6	±	3.0	24.0	±	4.2	0.442	0.482	0.593	0.211

Note: BMI, body mass index. Data are shown as mean ± standard errors.

**TABLE 2:** Scar information of subjects on NTP magnitudes.

		Magnitude		
		−105 mmHg (%)	−125 mmHg (%)	−145 mmHg (%)
Type of injury				
Surgeries		63.6	61.5	8.3
Abrasions		27.3	30.7	58.4
Burns		9.1	7.6	25.0
Insect bites		0.0	0.0	8.3
Location of injury				
torso		18.2	15.4	8.3
upper limbs		36.4	46.1	75.0
lower limbs		45.5	38.5	16.7

Note: This study included 11 subjects in the −105 mmHg magnitude, 13 subjects in the −125 mmHg **magnitude**, and 12 subjects in the −145 mmHg magnitude groups.

### 3.2 Effect of air insole on PGA

There is a significant decrease in scar thickness in all three NPT magnitudes. However, in the −125 mmHg magnitude, the viscoelasticity of the overall soft tissue increases significantly (Table 3). In the −125 mmHg group, the ***E2*** and ***E3*** post-treatment viscoelasticity significantly increased compared to pre-cupping. Based on the paired *t*-test, the effect of −105 mmHg magnitude significantly decreased thickness (3.7 ± 0.2 mm vs. 2.8 ± 0.1 mm, *p* < 0.001) between pre- and post-treatment. The effect of −125 mmHg magnitude also significantly decreased thickness (4.3 ± 0.5 mm vs. 3.9 ± 0.5 mm, *p* < 0.001), E2 had a significant increase (64.3 ± 12.9 kPa vs. 99.6 ± 18.1 kPa, *p* = 0.032), and E3 had a significant increase (75.8 ± 13.9 kPa vs. 125.5 ± 19.2 kPa, *p* = 0.009) between pre- and post-treatment. The effect of −145 mmHg magnitude significantly decreased thickness (3.5 ± 0.3 mm vs. 2.9 ± 0.2 mm, *p* = 0.001).

**TABLE 3:** Statistical results of the paired *t*-test with pre- and post-treatment.

Magnitude (mmHg)	Factor	Treatment						Paired *t*-test	
		Pre-treatment (mean ± SE)			Post-treatment (mean ± SE)			*p*-value	
105	Thickness (mm)	3.7	±	0.2	2.8	±	0.1	0.000	**
	E1 (N/mm^2^)	51.2	±	21.5	72.1	±	26.9	0.159	
	E2 (N/mm^2^)	54.4	±	21.3	85.2	±	30.4	0.097	
	E3 (N/mm^2^)	57.9	±	21.0	93.5	±	30.7	0.082	
125	Thickness (mm)	4.3	±	0.5	3.9	±	0.5	0.000	**
	E1 (N/mm^2^)	52.6	±	10.6	77.4	±	18.9	0.144	
	E2 (N/mm^2^)	64.3	±	12.9	99.6	±	18.1	0.032	*
	E3 (N/mm^2^)	75.8	±	13.9	125.5	±	19.2	0.009	**
145	Thickness (mm)	3.5	±	0.3	2.9	±	0.2	0.001	**
	E1 (N/mm^2^)	42.1	±	13.2	81.0	±	20.8	0.146	
	E2 (N/mm^2^)	86.2	**±**	29.4	120.9	±	23.6	0.411	
	E3 (N/mm^2^)	114.4	±	31.0	163.8	±	25.8	0.273	

Note: E1, approximately 5% of the initial thickness of the soft tissues as superficial layer viscoelasticity; E2, approximately 10% of the initial thickness of the soft tissues as medium layer **viscoelasticity**; E3, approximately 15% of the initial thickness of the soft tissues as deep layer viscoelasticity. Data are shown as mean ± standard errors; * significant difference (*p* < 0.05); ** significant difference (*p* < 0.01).

### 3.3 Effect of walking duration on the PPG and PGA

**FIGURE 8:**
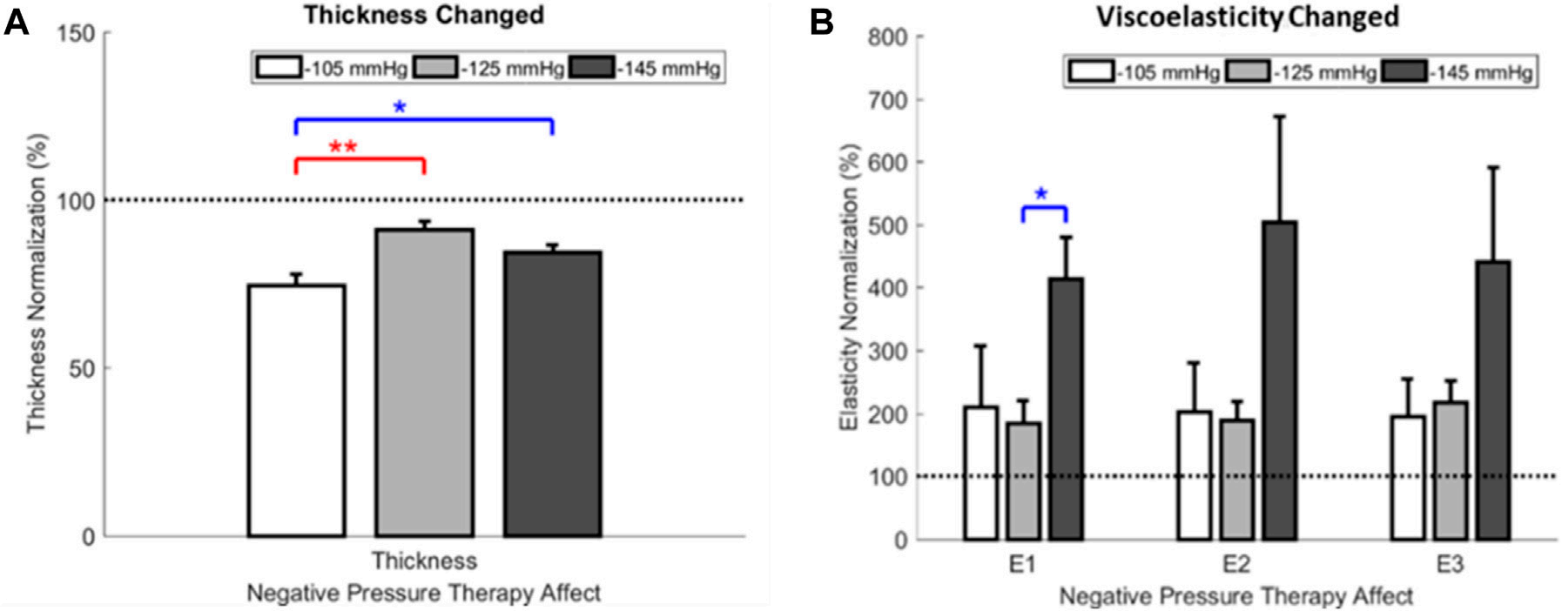
The ratio of thickness and viscoelasticity for NPT pre- and post-treatment. **(A)** Effect of NPT magnitudes on thickness. **(B)** Effect of NPT magnitudes on viscoelasticity. ***E1***, approximately 5% of the initial tissue thickness as the toe region’s viscoelasticity; ***E2***, approximately 10% of the initial tissue thickness as the heel region’s viscoelasticity; ***E3***, approximately 15% of the initial tissue thickness as the linear region’s viscoelasticity. **NPT**, negative pressure therapy.

Based on the one-way ANOVA, the effect of −105 mmHg magnitude on thickness significantly differed from the −125 mmHg (74.5 ± 3.3 percentage ratio vs. 91.3 ± 2.2 percentage ratio, *p* < 0.001) and −145 mmHg (74.5 ± 3.3 percentage ratio vs. 84.4 ± 2.2 percentage ratio, *p* = 0.010). The viscoelasticity of the ***E1*** showed significant differences between the −125 mmHg and −145 mmHg magnitudes (184.0 ± 36.0 percentage ratio vs. 413.7 ± 65.6 percentage ratio, *p* = 0.019) (Figure 8; Table 4).

**TABLE 4:** ANOVA Effect of negative pressure magnitude effect on the scar.

	Magnitude									One-way ANOVA *p-value*		Fisher least square difference (LSD) post hoc					
	−105 mmHg (mean ± SE)			−125 mmHg (mean ± SE)			−145 mmHg (mean ± SE)					−105 mmHg vs. −125 mmHg		−105 mmHg vs. −145 mmHg		−125 mmHg vs. −145 mmHg	
Thickness	74.5	±	3.3	91.3	±	2.2	84.4	±	2.2	0.001	**	0.000	**	0.010	*	0.052	
Elastic *E1*	209.7	±	96.7	184.0	±	36.0	413.7	±	65.6	0.032	*	0.822		0.052		0.019	*
Elastic *E2*	202.3	±	77.0	188.3	±	29.5	503.7	±	167.7	0.203		0.954		0.171		0.122	
Elastic *E3*	194.8	±	59.4	217.9	±	33.2	440.9	±	149.3	0.312		0.914		0.208		0.217	

Note: *E1*, approximately 5% of the initial thickness of the soft tissues as superficial layer viscoelasticity; *E2*, **approximately** 10% of the initial thickness of the soft tissues as medium layer viscoelasticity; *E3*, approximately 15% of the initial thickness of the soft tissues as deep layer viscoelasticity. Data are shown as mean ± standard errors; * significant difference (*p* < 0.05); ** significant difference (*p* < 0.01).

### 3.4 Correlation between the PPP, PPG, and PGA

There was a significant correlation between the ***E1***, ***E2***, and ***E3*** for all NPT magnitudes (r = 0.99–0.866, *p* < 0.05). However, there was no correlation between viscoelasticity and thickness ratio change. ***E1*** and ***E2*** showed a high correlation in three pressure magnitudes (r = 0.99–0.91, *p* < 0.01). ***E1*** and ***E3*** were highly correlated (r = 0.97–0.87, *p* < 0.01) in the −105 and −125 mmHg pressure magnitudes and were moderately correlated (r = 0.66, *p* < 0.05) in the −145 mmHg pressure magnitude. ***E2*** and ***E3*** were highly correlated in three pressure magnitudes (r = 0.99–0.88, *p* < 0.01) (Table 5).

**TABLE 5:** Correlation coefficients among the ***E1***, ***E2***, and ***E3*** in the three NPT magnitudes.

Parameter	Magnitude								
	−105 mmHg			−125 mmHg			−145 mmHg		
	Correlation	*p*-value		Correlation	*p*-value		Correlation	*p*-value	
Thickness & *E1*	0.20	0.537		−0.30	0.327		−0.10	0.735	
Thickness & *E2*	0.21	0.516		−0.14	0.638		−0.19	0.538	
Thickness & *E3*	0.25	0.440		−0.09	0.778		−0.23	0.443	
*E1* & *E2*	0.99	0.000	**	0.94	0.000	**	0.91	0.000	**
*E1* & *E3*	0.97	0.000	**	0.87	0.000	**	0.66	0.014	*
*E2* & *E3*	0.99	0.000	**	0.98	0.000	**	0.88	0.000	**

Note: The relationship between compression and deformation determines viscoelasticity. *E1*, approximately 5% of the initial thickness of the soft tissues as superficial layer viscoelasticity; *E2*, approximately 10% of the initial thickness of the soft tissues as medium layer viscoelasticity; *E3*, approximately 15% of the initial thickness of the soft tissues as deep layer viscoelasticity. Data are shown as mean ± standard errors; * significant difference (*p* < 0.05); ** significant difference (*p* < 0.01). NPT, negative pressure therapy.

## 4 Discussions

This study had three important findings: NPT reduced scar thickness and increased viscoelasticity. Furthermore, NPT could effectively increase scar viscoelasticity in *E2* and *E3* in the −125 mmHg group. Finally, after NPT, the correlation between the *E1* and *E3* elasticity was reduced in the −145 mmHg group.

This study’s first finding supports the hypothesis that NPT reduces scar thickness and increases viscoelasticity (Figure 8). NPT may reduce scar thickness by releasing scar contractures (Wilkes et al., 2012). Furthermore, external mechanical stimulation from NPT could release scar contractures by altering collagen compliance and aligning scar collagen fibers (Xu and Lu, 2011; Weidenhamer and Tranquillo, 2013). NPT external mechanical stimulation could also increase scar by improving tensile strength (Corr and Hart, 2013). The results are similar to those of current studies (Moortgat et al., 2016).

The increase in viscoelasticity may also be due to negative pressure promoting oxygenated hemoglobin to flood into the treatment site and increase blood volume (Li et al., 2023). However, the scar elastin is usually avascular (Franzeck et al., 1984; Almine et al., 2012), which would result in a difficult flow of blood into the avascular scar tissue, so the viscoelasticity appears unchanged after −125 mmHg treatment in ***E1*** (Joodaki and Panzer, 2018). This contention conforms to the research that demonstrated in animal experiments that an NPT magnitude of −125 mmHg can increase blood volume more than other magnitudes (Borgquist et al., 2011). Therefore, the increasing viscoelasticity is noticeable in the ***E2*** and ***E3*** as blood volume increases, and increased blood volume could stimulate endothelial proliferation and angiogenesis, which in turn promotes the growth of capillaries in scar tissue (Chen et al., 2005) and actuate scar fibers, which would then grow faster to heal.

The second finding was that the scar viscoelasticity ratio showed −145 mmHg significantly increased the response of elastin fibers’ structural (***E1***) more than the resistance of elastin fibers stretch (***E2***) after NPT (Figure 8B). There was no sign in the ***E2*** and the sliding of elastin or collagen (***E3***) (Fratzl and Weinkamer, 2007; Aziz et al., 2016; Joodaki and Panzer, 2018; Sharabi, 2022). Negative pressures between −125 and −145 mmHg may reach a critical point for the mechanical properties of the scar. An analysis of correlations was performed in this study to examine the changing factors of the NPT magnitudes. The scar viscoelasticity ratio changed after NPT and showed a significantly increased ***E1*** in the −125 mmHg and −145 mmHg treatment groups. There was no indication of change in the ***E2*** and ***E3*** values. This result may indicate that different NPT magnitudes have different effects on elastin structure (Joodaki and Panzer, 2018). However, there should be both sliding and realignment effects on elastin and collagen exist in ***E2*** and ***E3*** (Aziz et al., 2016; Sharabi, 2022). Because scarring usually occurs on the surface of the skin, negative pressures between −125 and −145 mmHg may reach a critical point for the mechanical properties of the scar. An analysis of correlations was performed in this study to examine the changing factors of the NPT magnitudes.

This study’s third finding is the correlation between E1 and E3 under NPT magnitude −145 mmHg. In a correlation test, we further explored differences in the scar viscoelasticity of different soft tissues. Their correlation is significant because the E1, E2, and E3 viscoelasticities belong to the same soft tissue category. Interestingly, the correlation coefficients between the ***E1*** and ***E3*** viscoelasticities were significantly reduced to 0.66 (moderate) in the −145 mmHg treatment, while they were more than 0.85 (very strong) in the other two magnitudes.

According to the correlation of NPT magnitudes, the viscoelasticity of the elastin stretch (***E1***) compared with the viscoelasticity of the effect of sliding and the alignment of collagen and elastin (***E3***) decreased in the −145 mmHg group (Gupta et al., 2010; Aziz et al., 2016; Sharabi, 2022).

**FIGURE 9:**
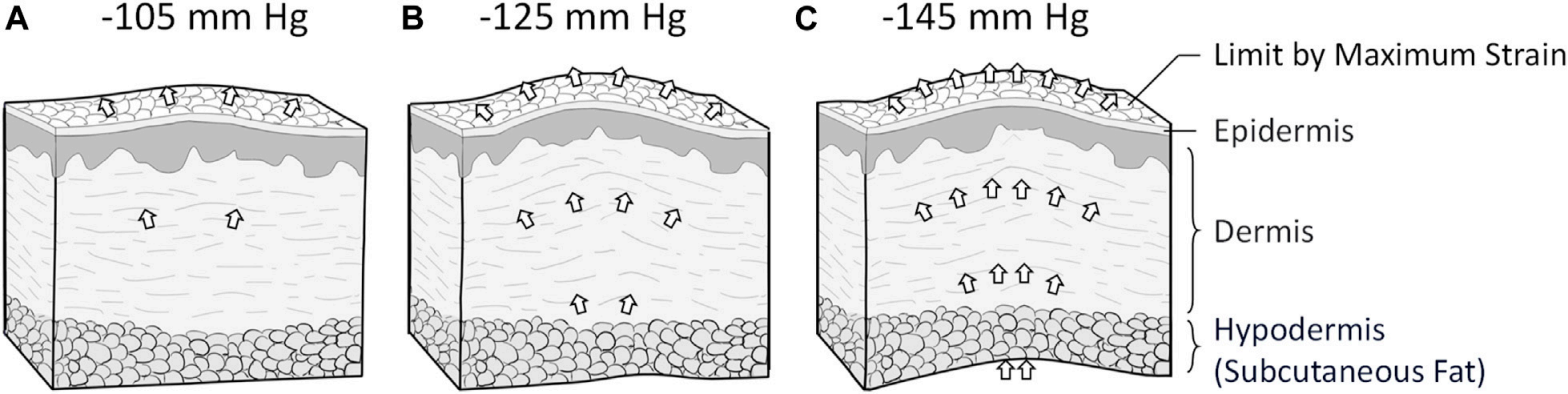
The different NPT magnitudes affect tissue viscoelasticity. **(A)** NPT magnitudes of −105 mmHg interfere with scar tissue’s superficial and heel regions. **(B)** NPT magnitudes of −125 mmHg interfere with superficial, medium, and deep scar tissues. **(C)** NPT magnitudes of −145 mmHg may interfere more effectively with deep soft tissue. It probably extends beyond the upper limit of the toe region of the soft tissue, so it does not show more extension than magnitudes of −125 mmHg NPT.

In the test of scar viscoelasticity, the soft tissue may have an elongation limit. When it reaches its limit, it can no longer extend or damage itself (Hendriks, 1969). The elastin fiber represented by ***E1*** exceeds its maximum elongation limitation when stretched during the elastic test and does not follow changes caused by a negative pressure change. A decline in correlation occurs because ***E1*** and ***E3*** have not yet reached the extension limit and still follow the effect caused by negative pressure. The mechanism is shown in Figure 9. Thus, although the magnitude at −145 mmHg showed a higher trend toward elastic improvement, to avoid rupture of superficial scar tissue or capillaries caused by negative pressure, we believe that −145 mmHg should not be used in clinical therapy (Zwanenburg et al., 2021).

**FIGURE 10:**
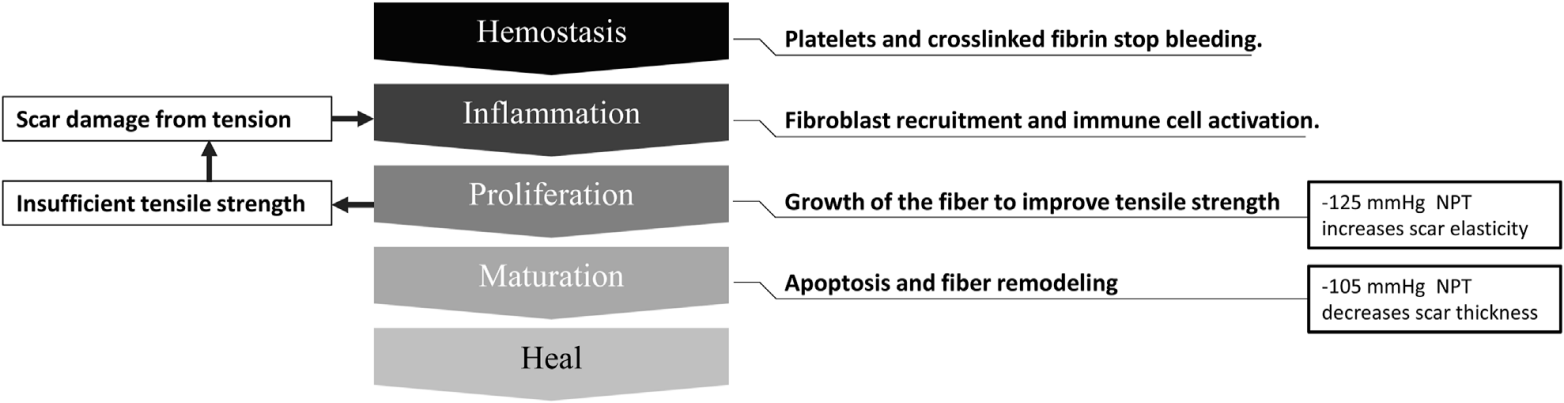
Scar healing phase and negative pressure intervention suggestions. NPT, negative pressure therapy.

Further scar developments may require different restoration effects (Gauglitz et al., 2011). Therefore, when prescribing NPT for scars, this study may help determine the appropriate magnitude. When scar fibers are maturing, the −105 mmHg magnitude therapy could facilitate their realignment (Corr and Hart, 2013). The proliferation stage may benefit from a negative pressure magnitude of −125 mmHg to increase their tensile strength (Iheozor-Ejiofor et al., 2018). The mechanism and clinical advice of the scar healing stage is shown in Figure 10.

There are some limitations in this study. The first limitation is that despite describing the post-treatment effects of different NPT magnitudes in this study, therapy of a longer duration may have different effects (Shen et al., 2022). As noted, scar growth typically spans approximately 1 year (Feng et al., 2020). Because our treatment tracking data were only collected once, they may not be adequate to capture this phenomenon fully. Furthermore, as the negative pressure increases, the therapy duration may induce different physiological responses (Lowe, 2017). Although 10 min is a valid NPT duration, longer NPT seems to have a greater clinical impact (Wang et al., 2020). Future studies in long-term follow-up scar populations and the effect of different therapy durations (10/20 min) are needed to improve clinical benefit.

Another limitation is that one-way ANOVA and paired t-tests were used for analysis in this study. However, the two- and three-way ANOVAs offer significant analytical power for stress–strain experiments. Future analyses could benefit from incorporating these statistical techniques as standard methods. Taking a broader view of stress–strain analysis may enhance the robustness and depth of our findings, leading to more nuanced insights and avenues for future research to improve statistical power. Another limitation of this study is the possible potential intervention of scar viscoelasticity, although it passed the reliability test. Surrounding undisturbed scar tissue was used for comparison.

The other limitation of this study is that even the viscoelastic recording method used in this study was adapted from a previous study (Zheng Y. and Mak A. J. J. O. B. E., 1999). This study also describes the post-treatment effects of different NPT magnitudes. However, the age of the subjects cannot be ruled out (Vexler et al., 1999), and the subject’s innate scar tissue viscoelasticity may interfere when measuring. Comparing the surrounding undisturbed scar tissue and treatment scar tissue of the same subjects may be a solution to this limitation in the future. Overall, this study is the first phase of our research to determine the effect of NPT treatment on scar tissue recovery and investigate potential effective therapeutic magnitudes. We intend to expand the subjects in the future to classify scar factors such as scar size, scar duration, and the cause of injury (Goodarzi et al., 2020). We also intend to consistently classify the different types of scars as immature, mature, atrophic, hypertrophic, or keloid (Mustoe and technologies, 2020).

## 5 Conclusion

In comparing the absolute changes pre- and post-treatment using negative pressure on the scar, our result showed that scar thickness significantly decreased in all negative pressure magnitudes, and the magnitude of −105 mmHg is the most significant, followed by −125 and −145 mmHg. The viscoelasticity of the scar was significantly increased in the −125 mmHg magnitude but not in the −105 and −145 mmHg magnitudes. Our findings support the hypothesis that the NPT magnitude change may contribute to the therapeutic effect. Overall, we present an effective −125 mmHg magnitude recommendation for improving scar viscoelasticity that can be applied to clinical practice. This study followed scientific research practices and proved that NPT could potentially treat scars, leading to more advances and new treatments.

## Data Availability

The raw data supporting the conclusions of this article will be made available by the authors, without undue reservation.
